# Editorial: Late-onset depression and mania: Diagnosis, treatment and life events as risk factors

**DOI:** 10.3389/fpsyt.2022.980366

**Published:** 2022-08-02

**Authors:** Delfina Janiri, Gabriele Sani, Mirko Manchia

**Affiliations:** ^1^Department of Psychiatry, Fondazione Policlinico Universitario Agostino Gemelli IRCCS, Rome, Italy; ^2^Department of Psychiatry and Neurology, Sapienza University of Rome, Rome, Italy; ^3^Section of Psychiatry, Department of Neuroscience, Università Cattolica del Sacro Cuore, Rome, Italy; ^4^Section of Psychiatry, Department of Medical Sciences and Public Health, University of Cagliari, Cagliari, Italy; ^5^Department of Pharmacology, Dalhousie University, Halifax, NS, Canada

**Keywords:** depression, mania, bipolar disorder, elderly, geriatric (aging)

Late-onset depression and mania are associated with frailty, impaired functioning, and 1.5- to 2-fold higher mortality risk (1–3). There is much debate in literature on the validity of late age at onset as a diagnostic distinction in geriatric patients. This issue is at odd with the idea of possible age-associated effects on early-onset illness. In other words, patients with late-life mood disorders are a heterogeneous group, including individuals with late-onset depression and mania, in whom the initial mood episode occurs in the old age, and individuals who had already experienced previous mood episodes earlier in life. Indeed, the pathophysiology of late-life mood disorders is multifactorial and complex. Recent findings suggest that microvascular dysfunction may be involved in the development of late-onset depression (4). In fact, the latter often cooccurs with other clinical symptoms that may share a microvascular origin (i.e., apathy and cognitive impairment). Similarly, a recent review showed that a first manic episode with an onset at over the age of 50 years appears to be strongly associated with underlying organic illness (2).

The recognition and accurate diagnosis of late-life mood disorders is challenging. The assessment of mood symptoms in elderly patients should focus on mood, anxiety, and cognitive symptoms as well as on the assessment of comorbidities, use of new medications and underlying neurobiological mechanisms. As above mentioned, an important point that still needs to be addressed is whether late-onset depression and mania can be considered as a distinct psychopathological entity from non-late-onset mood disorders. In the current Research Topic, (5) attempted at disentangling this particular issue, assessing clinical, psychopathological and temperamental features in a sample of Italian individuals aged >50 years old with late-onset bipolar disorders (BD) or non-late-onset BD. The authors found that late onset BD was associated with BD type II diagnosis, depressive episodes, mixed states, and predominant depressive and anxious affective temperaments. Conversely, non-late-onset BD presented with higher endocrinological and metabolic comorbidity, a more frequent diagnosis of BD type I, more manic episodes, and predominant hyperthymic affective temperament. One strength of this study was the exclusion of patients with cognitive impairment, dementia, psychiatric comorbidities and substance use disorders, thus limiting the potential heterogeneity of the sample. Authors provided initial evidence supporting a different psychopathological and clinical characterization of patients with late-onset BD. Affective temperaments in particular, defined as the basic predisposition to a level of activity, affective tone, or mood, may be considered as an early identifying predictor of late-onset BD. Future studies are needed to confirm these initial observations.

Most of the studies included in the current Research Topic focused on diagnosis, risk factors and prevention strategies of depression in the elderly. Regarding the importance of diagnostic reliability, (6) aimed at identifying distinct profiles of depressive symptoms in Chinese individuals aged ≥45. They used latent profile analysis to determine the optimal cutoff point for the 10-item Center for Epidemiologic Studies Depression Scale (CES-D-10), finding a three-profile solution as the best fit. They identified three clinical profiles, including minimal depression, mild depression, and moderate-severe depression. (7) applied machine learning approaches to identify risk factors for depression in home-based elderly Chinese aged ≥45. The study capitalized on a large sample size and a longitudinal design, using data from a nationally representative cohort study. Authors found that life satisfaction and cognitive ability were strongly associated with the prediction of future depression. Furthermore, a systematic review and meta-analysis by (8) focused on involuntary retirement as a specific risk factor for depression. Authors included 8 studies and found a significant association between involuntary retirement and depression, with a pooled RR of 1.31 (95% CI, 1.13–1.51; *P* = 0) for unexpected retirement compared to employment. Authors speculated that involuntary retirement may be specifically associated with changes in life patterns and social support, which have been shown as important predictors of depression. Interestingly, Orsolini et al. found that individuals with late-onset BD were more frequently retired than patients with a history of mood disorders. These findings may imply that retirement could represent a trigger risk factor in those individuals presenting with psychiatric vulnerability or a previous attenuated form of mood disorder. On the other hand, (9) investigated prevention strategies in Chinese individuals aged ≥65 years old. The sample, balanced for gender, included 6,287 elderly adults. They found a positive association between participation in social/sports groups, community-related organizations and better mental health. In their sample, they also found women to be more at risk for depressive symptoms than men. These data are consistent with what is available in the literature about the higher prevalence of mood disorders and psychological distress in the elderly female population (10, 11).

Last, a study included in the current Research Topic examines health literacy among Chinese outpatients aged ≥60 years old (12). In particular, authors assessed participants' awareness of depression and mania. They found that 86.1 and 36.4% of participants had heard of depression and mania, respectively. Authors highlighted that older individuals attending community healthcare services had good depression literacy but relatively poor mania literacy. They suggested that there is a strong need of designing public education programs for the aged population on mood disorders, in particular on manic symptoms.

The primary focus of this Research Topic was to present an overview of late-onset depression and mania, providing data on diagnosis, risk factors and treatment strategies. Despite the number of papers included in the current issue cannot be considered fully representative, results suggest that the late-onset mood disorders field is rapidly expanding. A critical point emerging from this overview is the need to establish a clear cutoff age for late-onset depression and mania. The studies, in fact, adopted different cut-off ages, ranging from ≥45 years old to ≥65 years old. This heterogeneity reflects the uncertainty expressed by the scientific community on this particular topic. In conclusion, there is clear evidence of the importance of a more precise characterization of late-onset depression and mania in geriatric patients. New effective interventions and prevention strategies of mood disorders in older individuals need to be developed, which requires a better understanding of the neurobiological mechanisms underlying depression and mania in this age group.

## Author contributions

DJ and MM wrote the first draft of the manuscript. GS critically revised the manuscript and provided important intellectual contributions. All authors have read and approved the final version.

## Conflict of interest

The authors declare that the research was conducted in the absence of any commercial or financial relationships that could be construed as a potential conflict of interest.

## Publisher's note

All claims expressed in this article are solely those of the authors and do not necessarily represent those of their affiliated organizations, or those of the publisher, the editors and the reviewers. Any product that may be evaluated in this article, or claim that may be made by its manufacturer, is not guaranteed or endorsed by the publisher.
